# Changing Molecular Profiles of Human Cryptosporidiosis Cases in Scotland as a Result of the Coronavirus Disease, COVID-19 Pandemic

**DOI:** 10.3389/bjbs.2023.11462

**Published:** 2023-08-28

**Authors:** Ross Bacchetti, Lisa Connelly, Lynda Browning, Claire L. Alexander

**Affiliations:** ^1^ Scottish Microbiology Reference Laboratories (Glasgow), Glasgow Royal Infirmary, Glasgow, United Kingdom; ^2^ Clinical and Protecting Health Directorate, Public Health Scotland, Glasgow, United Kingdom

**Keywords:** human cryptosporidiosis, molecular variants, COVID-19 pandemic, Scotland, *Cryptosporidium*

## Abstract

*Cryptosporidium*, the most frequently reported parasite in Scotland, causes gastrointestinal illness resulting in diarrhoea, nausea and cramps. Two species are responsible for most cases: *Cryptosporidium hominis (C. hominis)* and *Cryptosporidium parvum* (*C. parvum*). Transmission occurs faecal-orally, through ingestion of contaminated food and water, or direct contact with faeces. In 2020, the COVID-19 pandemic led to global restrictions, including national lockdowns to limit viral transmission. Such interventions led to decreased social mixing, and reduced/no local and international travel, which are factors associated with transmission of multiple communicable diseases, including cryptosporidiosis. This report assessed the impact of the pandemic on Scottish cryptosporidiosis cases, and identified changes in circulating molecular variants of *Cryptosporidium* species. Molecular data generated using real time PCR and GP60 nested-PCR assays on laboratory-confirmed cryptosporidiosis cases reported during 2018–22 were analysed. The Scottish Microbiology Reference Laboratories (SMiRL), Glasgow, received 774 *Cryptosporidium*-positive faeces during 2018–22, of which 486 samples were successfully subtyped. During this time period, *C. hominis* (*n* = 155; 21%) and *C. parvum* (*n* = 572; 77%) were the most commonly detected species. The total number of cases during 2020, which was greatly affected by the pandemic, was markedly lower in comparison to case numbers in the 2 years before and after 2020. The most predominant *C. hominis* family detected prior to 2020 was the Ib family which shifted to the Ie family during 2022. The most common *C. parvum* variant during 2018–22 was the IIa family, however a rise in the IId family was observed (*n* = 6 in 2018 to *n* = 25 in 2022). The dominant *C. hominis* subtype IbA10G2, which accounted for 71% of *C. hominis* subtypes in 2018–19 was superseded by three rare subtypes: IeA11G3T3 (*n* = 15), IdA16 (*n* = 8) and IbA9G3 (*n* = 3) by 2022. Frequently reported *C. parvum* subtypes in 2018–19 were IIaA15G2R1 and IIaA17G1R1, accounting for 59% of total *C. parvum* subtypes. By 2022, IIaA15G2R1 remained the most common (*n* = 28), however three unusual subtypes in Scotland emerged: IIdA24G1 (*n* = 7), IIaA16G3R1 (*n* = 7) and IIaA15G1R2 (*n* = 7). Continuous monitoring of *Cryptosporidium* variants following the pandemic will be essential to explore further changes and emergence of strains with altered virulence.

## Introduction

*Cryptosporidium* is the most common parasitic aetiological agent of gastrointestinal infections reported in Scotland, United Kingdom [1]. The infective stage, known as the oocyst, is transmitted through ingestion of contaminated food or water, or by direct contact with infected faeces from human and/or animal sources [2]. Infection results in symptoms including diarrhoea, nausea, cramps, fatigue and weight loss, which are often self-limiting in the immunocompetent, however can be chronic and potentially life-threatening in the immunocompromised and young children [2]. Throughout the UK, the majority of human cryptosporidiosis cases are caused by two *Cryptosporidium* species; the anthroponotic *Cryptosporidium hominis* (*C. hominis*), and the zoonotic *Cryptosporidium parvum* (*C. parvum*) [3]. The oocysts of *C. parvum* and *C. hominis* appear morphologically identical by microscopy, therefore molecular-based detection methods are required to fully speciate this parasite [1, 4]*.* Over the past few decades, the ability to determine molecular variants of *Cryptosporidium* at a genotypic level has become possible using sophisticated laboratory tools. Most recently, a multi-locus variable number of tandem repeats analysis (MLVA) scheme has been developed of high discriminatory power which has yet to be implemented globally [5]. The majority of specialist laboratories currently amplify a parasite-specific 60 kDa glycoprotein gene (GP60), to determine the barcode of this hypervariable microsatellite region when subtyping *Cryptosporidium* [6]. Possessing the ability to subtype this parasite has proven to be indispensable for monitoring circulating molecular variants within communities, and managing local, national and international outbreaks.

The onset of the Coronavirus disease, COVID-19 pandemic caused by the SARS-CoV-2 virus, resulted in sudden, major change to everyday life on a global scale, with the first confirmed COVID-19 case in Scotland occurring on 1st March 2020 [7]. The UK and Scottish Governments applied measures most notably, regional and national lockdowns, in a bid to impede viral transmission. By 29th May 2020, Scotland began its route map out of lockdown, which over time, permitted pre-pandemic activities to gradually recommence. However further shorter lockdowns were implemented both at a regional and national level in response to rising COVID-19 levels [7]. Implementation of such measures resulted in decreased social mixing, closure of non-essential businesses such as food and drink venues, a ban on international travel, and improved hand hygiene, all of which had the potential to impact on the number of cases, and molecular subtypes of *Cryptosporidium* within the population.

This report highlights the changing dynamics of *Cryptosporidium* at a molecular level from 2018–22 in Scotland to gain a better understanding of the impact of COVID-19 restrictions and human cryptosporidiosis.

## Materials and Methods

During 2018–22, a total of 2063 cases of laboratory-confirmed cryptosporidiosis were reported to Public Health Scotland (PHS) (5-year average *n* = 611; range 502–786 cases; personal communication). Of those laboratory-confirmed cases, a total of 744 faecal samples positive for *Cryptosporidium* oocysts, antigen or DNA were received at the Scottish Microbiology Reference Laboratories (SMiRL) Glasgow, UK for molecular analysis. Faeces were sent from National Health Service (NHS) Diagnostic Microbiology Laboratories across Scotland as part of the Scottish Government-funded *Cryptosporidium* Surveillance and Outbreak Service. As funding was not available to subtype every positive sample, a selection of faeces from rural, semi-rural and urban areas were subtyped to give a useful indication of circulating types within Scotland. Multiple samples received from the same case within a 4-week period were classed as one sample.

Samples were transported and stored at room temperature. On arrival, faeces were concentrated using Parasep concentrators (Apicor, Berkshire, United Kingdom), and *Cryptosporidium* DNA extracted on the automated NucliSENS EasyMag platform (BioMerieux, Basingstoke, United Kingdom). To determine if samples were *C. hominis* or *C. parvum*, a previously described real-time PCR assay was employed using the Light Cycler^®^ 480 platform (Roche Diagnostics, West Sussex, United Kingdom) [8]. The molecular profile of each sample was assigned by amplifying a region of the GP60 gene followed by bi-directional sequencing on the Applied Biosystems 3500XL (ThermoFisher Scientific, Inchinnan, United Kingdom), as previously described [1]. Sequences were aligned to reference databases (EMBI and NCBI BLASTn) and repeat sequences determined manually.

## Results

Of the 744 faecal DNA samples speciated, the majority were found to be either *C. hominis* (*n* = 155; 21%) or *C. parvum* (*n* = 572; 77%). Other species accounted for less than 3% of the total (*C. cuniculus n* = 5; *C. ubiquitum n* = 6; *C. meleagridis n* = 2; *C. canis n* = 1; *C. erinacei n* = 1; mixed infection with *C. parvum* and *C. hominis n* = 2).

**FIGURE 1:**
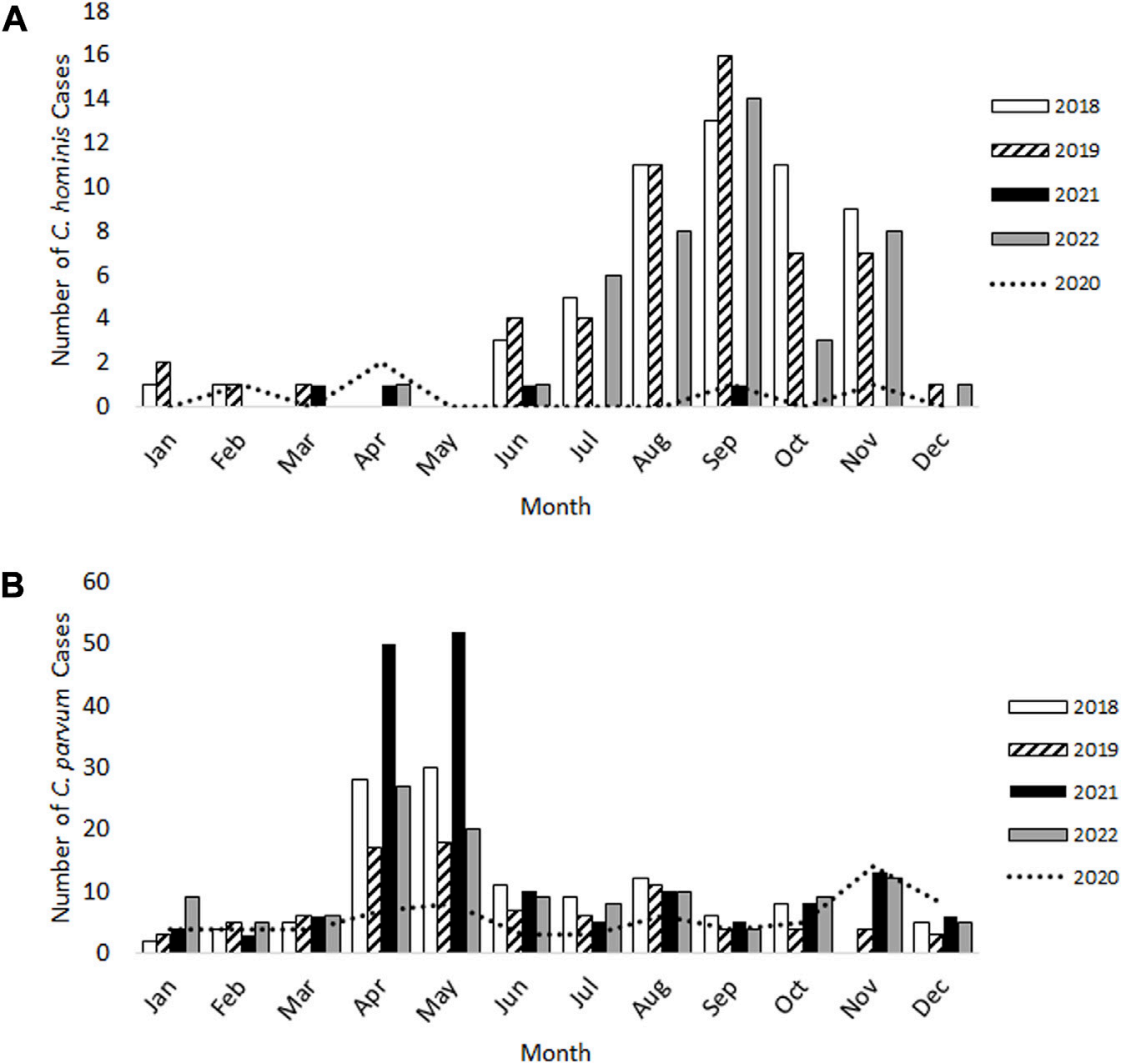
Laboratory-confirmed cases of *Cryptosporidium hominis* **(A)** and *Cryptosporidium parvum* **(B)** in Scotland from 2018–22.

For *C. hominis*, a total of 54 cases were identified in 2018 and a further 54 cases in 2019, with the expected late summer/autumn rise observed in September (Figure 1A). In 2020 and 2021 which were greatly impacted by lockdowns and restrictions, only five cases were identified with no autumn peak evident. During 2022, when COVID-19 lockdowns and restrictions had ended, the total number of *C. hominis* cases (*n* = 42) were similar to pre-pandemic levels (Figure 1A).

For *C. parvum*, 120 cases and 88 cases were reported during 2018 and 2019 respectively, with the expected rise occurring over springtime (April-May) and again in August (Figure 1B). Whilst fewer *C. parvum* cases were reported in 2020 (*n* = 70), an additional, small peak was observed in November 2020 (*n* = 14 cases), which had not occurred in the previous 2 years (Figure 1B). This increase was also evident during 2021 and 2022 (Figure 1B). In 2021, the total number of *C. parvum* cases was 172, where the expected springtime increase during April-May was observed, but the number of cases were 1.8-fold and 2.8-fold greater than those identified in the same months during 2018 and 2019, respectively. In 2022, 124 *C. parvum* cases were reported, and the spring peak occurred, similar to 2018 and 2019 (Figure 1B).

Of the 744 faecal DNA samples speciated, 486 (65%) were successfully subtyped. Sixty-eight (14%) were *C. hominis* subtypes, and 415 (85%) were *C. parvum* subtypes. A further three samples identified as *C. cuniculus* were successfully subtyped (data not shown).

There was a shift in the most predominant *C. hominis* subtype family where the Ib family was most frequently reported in 2018 (*n* = 29), with only one case each of the Ie and Id variants. However, by the end of 2022, only three cases of the Ib family were identified, compared to nine cases of the Id, and 15 of the Ie families (Table 1).

**TABLE 1:** Most commonly detected *Cryptosporidium hominis* and *Cryptosporidium parvum* molecular families from 2018–22.

	*C. hominis*			*C. parvum*		
Year	Ib	Id	Ie	Ila	Ilc	Ild
2018	29	1	1	62	1	6
2019	2	0	0	56	2	11
2020	1	0	0	30	2	19
2021	0	0	1	116	1	17
2022	3	9	15	65	2	25

Before the implementation of 2020 COVID-19 lockdowns and restrictions, a total of 25 cases of the common *C. hominis* IbA10G2 subtype were identified in 2018–19 (71% of total *C. hominis* subtyped in 2018–19). In 2020, only two cases of *C. hominis* were subtyped (IbA10G2 *n* = 1, IaA24R2 *n* = 1). During 2021–22, there were no reports of the common IbA10G2 subtype but the emergence of three rare subtypes in Scotland were documented; IeA11G3T3 (*n* = 15); IdA16 (*n* = 8) and IbA9G3 (*n* = 3) (Table 2). Of the 415 *C. parvum* samples successfully subtyped, the three most common molecular families were the IIa (*n* = 329), IId (*n* = 78) and the IIc (*n* = 8) (Table 1). Whilst the IIa family remained the most common family throughout 2018–22, of interest, there was a gradual increase in numbers of the IId family (Table 1). Of note, for *C. parvum*, the total number of different subtypes identified in the springtime peak (April-May) throughout 2018–22 varied. During 2018–2020, there was an average of eight different subtypes. However in 2021, the number of different subtypes increased to 18, and remained elevated in 2022 with 13 different subtypes identified.

**TABLE 2:** *Cryptosporidium hominis* and *Cryptosporidium parvum* subtypes detected from 2018–22.

		Year				
		2018	2019	2020	2021	2022
*C. hominis*	IbA10G2	23	2	1	0	0
	IeA11G3T3	1	0	0	1	15
	IbA12G3	5	0	0	0	0
	IaA24R2	0	0	1	0	0
	IdA16	0	0	0	0	8
	IbA9G3	0	0	0	0	3
*C. parvum*	IIaA16G2R1	0	1	0	1	1
	IIaA17G1R1	20	13	5	24	6
	IIaA15G2R1	30	19	15	54	28
	IIdA24G1	0	2	2	0	7
	IIaA18G2R1	2	1	1	3	0
	IIcA5G3	1	2	2	1	2
	IIaA13G1R2	0	1	0	5	1
	IIdA17G1	3	0	4	5	5
	IIaA21G4R1	0	5	1	0	0
	IIdA22G1	1	0	6	3	3
	IIaA19G1R1	1	0	0	5	0
	IIaA15G1R2	2	3	2	1	7
	IIaA16G3R1	1	1	1	1	7
	IIdA19G1	1	1	4	3	5

The most common subtypes in 2018–19 were IIaA15G2R1 (*n* = 30 and *n* = 19, 2018 and 2019, respectively) and IIaA17G1R1 (*n* = 20 and *n* = 13 in 2018 and 2019, respectively), which accounted for 59% of cases subtyped. In 2020, whilst IIaA15G2R1 remained the most frequently reported subtype (*n* = 15; 29% of all *C. parvum* subtypes), the second most common was a IId subtype, namely, IIdA22G1 (*n* = 6), which accounted for 12% of all *C. parvum* isolates typed that year. In 2021, the IIaA15G2R1 and IIaA17G1R1 subtypes were most frequently observed (*n* = 54 and *n* = 24, respectively), accounting for 40% and 18% of the total. However, during 2022, the IIaA15G2R1 remained the most common (*n* = 28; 30%), but numbers of more unusual subtypes IIdA24G1 (*n* = 7), IIaA16G3R1 (*n* = 7) and IIaA15G1R2 (*n* = 7) were similar to the common IIaA17G1R1 type (*n* = 6) (Table 2).

## Discussion

In this report, we highlight that during the past 5 years, *C. hominis* only represented around one-fifth of those cases that were successfully typed, with *C. parvum* accounting for almost 80%. This is in contrast to previously published data from Scotland during 2012–13 and unpublished data from 2014–17 (data not shown), where similar numbers of *C. hominis and C. parvum* cases were reported [1]. This change in recent years is likely to reflect the altered human behaviours as a result of the pandemic restrictions, which included improved hand hygiene, reduced social interactions, a reduction in dining out at food and drink establishments, and reduced or no local/international travel [7, 9].

Similar to published data [1], this study found that human cryptosporidiosis cases normally occurred seasonally, with a peak in late-Spring of mainly zoonotic *C. parvum* infections coinciding with the lambing/calving season and open farm outbreaks, and a second peak occurring in late-Summer/early-Autumn mainly comprising of *C. hominis* infections assumed to be associated with an increase in human to human transmission locally, and also international travel [10].

Further, more in-depth analysis of epidemiological data for each case is necessary to ascertain what exposure(s) were likely to have resulted in each infection, but with the demand for limited resources within Public Health Scotland to support and prioritise COVID investigations, this has not been possible in recent times.

During 2020, which was greatly impacted by restrictions and lockdowns implemented to limit transmission of COVID-19, cases of cryptosporidiosis were markedly lower which is a similar pattern for the incidence of other gastrointestinal (GI) illnesses in the United Kingdom [11, 12]. A recent report on the effects of COVID-19 restrictions on cryptosporidiosis cases in England and Wales described similar findings to that observed in Scotland, where the incidence of *C. hominis* and *C. parvum* decreased markedly from the onset of COVID-19 restrictions [13]. These finding differed to that of a study from New Zealand, which reported an absence of *C. hominis* cases within the first few months of COVID-19 restrictions, yet *C. parvum* cases continued to be reported at a similar level [14].

Indirect effects of the national lockdown and imposed COVID-19 restrictions are likely to have impacted on the observed decrease in cryptosporidiosis cases during 2020. The closure of all non-essential businesses, including those previously linked to cryptosporidiosis outbreaks, such as swimming pools, open farms, food outlets and restaurants, may have contributed to lower cases being observed during this time [9, 12, 15]. Improved hand hygiene was encouraged throughout the pandemic, which is also an important measure in minimising transmission of GI infections, and is therefore likely to have decreased human-to-human transmission [16]. Starting 29th May 2020, the route map out of lockdown commenced in Scotland, which gradually permitted more socialising and pre-pandemic activities. However during this period of returning to normality, regional lockdowns were implemented in areas of Scotland identified to have high numbers of COVID-19 cases [7]. On implementation of the route map out of lockdown, there was a general reluctance to travel which is likely to have impacted on the number of cases, especially *C. hominis* during late-Summer/early-Autumn of 2020 [17]. Perhaps the potential loss in confidence to book holidays for fear of cancellations, and the need to be fully vaccinated when travelling to certain areas, impacted on numbers not fully returning to pre-pandemic levels by 2022 [18]. Whilst this study reported a decrease in laboratory-confirmed cryptosporidiosis cases during 2020, the true number of cases within the community is likely to be under-represented as a result of the public being advised to only contact healthcare providers in emergencies. Although this alleviated pressures on General Practitioners (GPs) and hospitals during the pandemic, it is likely to have resulted in individuals being reluctant to contact GPs with gastrointestinal illness.

The large spike in *C. parvum* cases observed in April and May of 2021, is likely to reflect the reopening of non-essential businesses including swimming pools, food outlets, petting farms/open farms, which enabled mixing of groups of individuals, and increased exposure to infected humans and animals, especially direct contact with young animals.

Over the time period covered in this study, the most predominant *C. hominis* family detected changed from the Ib family, which is the most common *C. hominis* family in Europe [19], to the Ie family followed by the Id family, both of which are commonly found in low/middle income countries [19]. The three rare *C. hominis* subtypes identified, namely, IeA11G3T3, IdA16 and IbA9G3 were likely to have been imported to Scotland once international travel began after the easing of pandemic lockdowns. One of the three rare *C. hominis* variants, namely, IeA11G3T3, is associated with infection in Asia, Africa and South America, and was previously identified in Scotland, with epidemiological links to a contaminated swimming pool [1, 20–22]. Subtype IdA16 has been previously identified in England, with most recent infections associated with a swimming pool outbreak [23]. To our knowledge, this is the first account of the IdA16 subtype within the Scottish population. A recent study found IdA16 infections identified in Sweden were associated with travel from Sri Lanka and China [24]. Lastly, the rare IbA9G3 subtype has been reported from Malawi, Kenya and India [19]. It has been previously reported that detection of rare *C. hominis* subtypes is associated with international travel [25]. Therefore, detection of these rare *C. hominis* subtypes post-2020 within the Scottish population was likely associated with increased travel beyond Europe following the pandemic, although as international travel had not yet reach pre-pandemic levels, consideration for possible imported contaminated consumables should be given [18].

In this report, the most predominant *C. parvum* family was IIa, in particular, IIaA15G2R1 which has also been reported from calves and geese in Scotland [26, 27]. Of interest, there was an increase in the number of IId family cases which is found throughout Europe, and is associated with infection of ruminants, including sheep, goats and calves [19, 27]. Throughout the pandemic, an increase in outdoor exercise and leisure activities e.g., wild water swimming was observed. Spending more time in green spaces is likely to have resulted in greater exposure to farmed animals and/or their faeces, resulting in an increase in the incidence of infection with the IId family [28, 29]. This may have also contributed to the increase in number of different *C. parvum* subtypes observed between April-May in 2021 and 2022. One of the more unusual *C. parvum* subtypes commonly observed post-pandemic namely, IIdA24G1, was previously identified in European hedgehogs (*Erinaceus europaeus*), Spanish lambs and humans in Sweden and Australia [30–33]. Although of interest, to our knowledge, the IId family has not been identified from animals within Scotland (Personal communications, Moredun Institute, Scotland). There is the possibility that the emergence of the IId family in Scotland is via an imported consumable as certain variants including the IIdA24G1 subtype have been associated with European outbreaks involving salad items [34]. The rare IIaA16G3R1, has previously been associated with cattle infections [19], and IIaA15G1R2, has been reported from UK outbreaks involving open farms [35]. As only a proportion of *Cryptosporidium*-positive faeces are typed in Scotland, due to the limited funds available, combined with the presence of PCR inhibitors in faeces, only 44% of *C. hominis* positive samples and 73% of *C. parvum* positive samples were subtyped.

This work represents an advance in biomedical science because it highlights that *Cryptosporidium* continues to be a significant cause of gastrointestinal disease, and the COVID-19 pandemic has resulted in changes to both the dominant species and subtypes circulating within the Scottish population. It will be crucial to monitor the molecular variants over the coming years to see if these changes remain stable, and if new and emerging strains appear with altered virulence as we exist in the post-pandemic era.

## Data Availability

The datasets presented in this article are not readily available because Public Health Scotland are owners of the data. Enquires to access the data should be made to the corresponding author. Requests to access the datasets should be directed to ross.bacchetti@ggc.scot.nhs.uk.
